# Prevalence of allergic sensitization to *Platanus occidentalis* among adults with allergic rhinitis: A multicenter study

**DOI:** 10.5415/apallergy.0000000000000127

**Published:** 2023-12-18

**Authors:** Martín Bedolla-Barajas, Javier Domínguez-Morales, Ilse Mariana Loya-Barriga, Angie Bedolla-Pulido, Luis Alfredo Jiménez-Huerta, Jaime Morales-Romero

**Affiliations:** 1 Clinical Allergy and Immunology Department, New Civil Hospital of Guadalajara “Dr. Juan I. Menchaca,” Guadalajara, Mexico; 2 Clinical Allergy and Immunology Department, General Zone Hospital No. 1, Mexican Social Security Institute, Tapachula, Mexico; 3 Clinical Allergy and Immunology Department, Christus Muguerza Hospital, Chihuahua, Mexico; 4 University Center for Health Sciences, University of Guadalajara, Guadalajara, Mexico; 5 Public Health Institute, Veracruzana University, Xalapa, Mexico

**Keywords:** Allergic rhinitis, pollen

## Abstract

**Background:**

In the Americas there are few studies that have evaluated the frequency of allergic sensitization to *Platanus occidentalis* or sycamore pollen in adult patients with allergic rhinitis (AR).

**Objective:**

To determine the prevalence of allergic sensitization to *P. occidentalis* and to identify factors associated with its presentation.

**Methods:**

A cross-sectional study was carried out in 3 centers distributed in the northwest, west, and southeast of Mexico. Allergic sensitization to *P. occidentalis* was determined with a skin prick test. Prevalence and 95% confidence intervals (CI) were estimated.

**Results:**

A total of 404 patients were included, women were 233 (57.7%); the age mean was 33.8 ± 12.9 years. The overall prevalence of sensitization to *P. occidentalis* was 20.8% (95% CI, 17.1%–25.0%); in the northwestern: 15.9% (95% CI, 9.6%–25.1%); in the western: 21.8% (95% CI, 15.4%–29.9%); and in the southeastern: 22.4% (95% CI, 17.1%–38.8%). Multivariate analysis showed to the following allergens as factors associated with sycamore allergic sensitization: tree pollens (OR, 3.19; *P* = 0.001), weeds (OR, 2.49; *P* = 0.004), fungi (OR, 1.96; *P* = 0.014), and dog or cat epitheliums (OR, 1.88; *P* = 0.018).

**Conclusion:**

Allergic sensitization to *P. occidentalis* pollen in AR patients is not an infrequent event; consequently, we recommend doing the challenge test in all patients with this allergen, especially in those regions where the tree is present.

## 1. Introduction

Pollen from trees worldwide is among the main allergen sensitivity inducers; pollens from ash, oak, birch, cypress, etc. are found within this group.

Traditionally, the characteristics that pollen must meet in order to be considered as a cause of pollinosis are: (1) the plant should produce enough pollen quantity; (2) pollen should be present in the patient´s environment; (3) it should be produced in large quantities and be light enough as to be transported by the wind and, finally, (4) it should be able to produce an allergic response [1]. Pollen from *Platanus occidentalis*, fits perfectly to these qualities. It is also known as plane tree or sycamore and is native to North America and Europe; it could reach up to 30–50 m height with monoecious flowers [2, 3]. Sycamore is usually an ornamental plant in big cities’ sidewalks, not just for its beauty but also for its easy adaptability to polluted environments [4]. *Platanus* tree belongs to the order *Proteales;* where species like *P. occidentalis*, *P. orientalis*, and *P. acerifolia*, are among the most representative [3].

*Platanus* species are pollinated by wind [3]; and its pollen has been reported in several cities around the world, including Mexico [5, 6] flowers appear typically from March to May [3, 5, 7, 8].

Twenty-five years ago, plane tree pollen was recognized as a relevant allergen [9]; up to now, different studies worldwide have reported how often such pollen sensitizes patients with allergic diseases; depending on tree abundance in the environment, pollen exposure sensitization frequency goes from 1% to 57% of allergic rhinitis (AR) cases [7, 9-12].

In the Americas, no previous studies exist reporting the number of patients with AR sensitized to *P. occidentalis*. The present study aimed to determine the prevalence of allergic sensitization to *P. occidentalis* pollen in adults with AR from 3 allergology medical centers in Mexico. Additionally, associated factors to such sensitization were identified.

## 2. Methods

### 2. 1. Design

Through a cross-sectional, multicenter study that consecutively recruited adult patients with recent AR diagnosis from each allergology service of the participating centers. Asthma or atopic dermatitis patients were excluded. This study was carried out from August 2019 to March 2020.

### 2. 2. Setting

Three medical centers distributed in Mexican territory were included. Western region was represented by the university hospital in Guadalajara, Jalisco, and the second most populated city in Mexico; with a mean altitude slightly above 1,500 m from sea level. This hospital offers services to the general population, mostly without social security.

A second center was Hospital Christus Muguerza in Chihuahua, Chihuahua at Northeastern Mexico, with a mean altitude of 1,400 m above sea level; a medical unit that offers private services.

Finally, a secondary level hospital at the center Southeastern in Tapachula, Chiapas with an altitude of 117 m above sea level; Instituto Mexicano del Seguro Social, one of the main social security institutions that offers health services mostly to workers.

### 2. 3. Definitions

AR was defined by the presence of a personal history of at least 2 of the following: hyaline rhinorrhea; sneezes in salvos; nasal itching and nasal obstruction continuously for at least an hour and almost daily. It was classified as mild or moderately acute and intermittent or persistent according to symptom’s severity and frequency [13].

Oral allergy syndrome was assessed by the presence of oral itching, edema of the lips, tongue, or palate, appearing continuously after food intake [14].

Conventionally, allergic conjunctivitis was defined with the presence of ocular itching, redness or tearing [15], associated with AR symptoms.

### 2. 4. Skin prick test technique

Cutaneous tests were taken to all patients, and they were invited to discontinue the use of antihistaminics, steroids, and any other drug that could interfere with the results, for at least a week before the procedure. Cutaneous tests were performed with the technique used in each center. In general, each participating center included allergens to mite, cockroach, and epithelium as well as grass, weed, and tree pollen depending on each region; *P. occidentalis’* pollen was added in centers where it was not habitually applied. As positive and negative controls, histamine and glycerin or Evans’ solution, were used, respectively. Once again, each center’s researcher decided anatomical site for cutaneous tests. A cutaneous test was considered positive if papule sized 3 mm or more compared to the negative control, after 15 minutes of its application [16].

### 2. 5. Statistical analysis

In order to know the ratio of sensitive subjects to *P. occidentalis* pollen; the number of positive cases to such allergen was divided by the total of participants; the same procedure was performed in each center. Chi-square test was used to compare proportions. Statistical significance was assumed when *P* ≤ 0.05. Associated factors to sycamore sensitization were identified by binary logistic regression. IBM SPSS Statistics 20 (IBM Corp., Armonk, NY) program was used for statistical analysis.

### 2. 6. Ethics

All participants signed an informed consent and their right to be included was respected at any time. This research was approved by Ethic and Research Committee from Nuevo Hospital Civil de Guadalajara “Dr. Juan I. Menchaca” (Ethical Committee CEI-042-2019), Guadalajara, Mexico (Chairperson Aguilar J, MD) on July 1, 2019.

## 3. Results

Sample included 404 patients; 124 came from a western center, 192 from the southeastern and 88 from the northeastern of Mexico, (Table 1). Mean age was 34 years with a light prevalence of women (58%); AR began at a mean of 20 years old; symptoms intensity was mild-moderate in more than 80% and persistent in almost 90% of the cases. At the same time, oral allergy syndrome and allergic conjunctivitis were reported in 11% and 68% of the patients, respectively. Aeroallergens sensitivity among patients were mostly house dust mite and pollen from trees, and fungi in the least quantity.

**Table 1.** Clinical characteristics of patients with allergic rhinitis

	Center				*P*
	Total	Guadalajara (Western)	Tapachula (Southeastern)	Chihuahua (Northeastern)	
	n = 404	n = 124	n = 192	n = 88	
Age, years					
Mean ± SD	33.8 ± 12.9	32.2 ± 14.9	35.0 ± 11.2	33.6 ± 13.1	0.165
Median (P25–P75)	31 (23–43)	28 (20–42)	35 (27–43)	31 (23–41)	NA
Minimum–Maximum	16–89	16–85	17–89	16–81	NA
Sex, n (%)					
Woman	233 (57.7)	87 (69.9)	88 (45.8)	58 (65.9)	<0.001
Man	171 (42.3)	37 (29.8)	104 (54.2)	30 (34.1)	
Allergic rhinitis					
Age of onset, years					
Mean ± SD	20.3 ± 13.0	20.6 ± 14.3	6.6 ± 5.3	18.4 ± 12.1	<0.001
Median (P_25_–P_75_)	9 (5–17)	16 (10–27)	4 (5–7)	15 (10–25)	NA
Minimum–Maximum	1–64	1–64	2–59	1–61	NA
Severity, n (%)					
Mild	63 (15.6)	51 (41.1)	0 (0)	12 (13.6)	<0.001
Moderate-Severe	341 (84.4)	73 (58.9)	192 (100)	76 (86.4)	
Duration, n (%)					
Intermittent	53 (13.1)	38 (30.6)	0 (0)	15 (17.0)	<0.001
Persistent	351 (86.9)	86 (69.4)	192 (100)	73 (83.0)	
Atopic comorbidity, n (%)					
Oral allergy syndrome	45 (11.1)	31 (25.0)	2 (1.0)	12 (13.6)	<0.001
Allergic conjunctivitis	276 (68.3)	80 (64.5)	133 (69.3)	63 (71.6)	0.511
Allergic sensitization, n (%)					
Mites	261 (64.6)	79 (63.7)	146 (76.0)	36 (40.9)	<0.001
Cockroach mix	138 (34.2)	50 (40.3)	70 (36.5)	18 (20.5)	0.007
Cat or dog epitheliums	130 (32.2)	37 (29.8)	48 (25.0)	45 (51.1)	<0.001
Trees	259 (64.1)	55 (44.4)	132 (68.8)	72 (81.8)	<0.001
Weeds	242 (59.9)	45 (36.3)	128 (66.7)	69 (78.4)	<0.001
Grass	237 (58.7)	51 (41.1)	115 (59.9)	71 (80.7)	<0.001
Fungi	107 (26.5)	22 (17.7)	64 (33.3)	21 (23.9)	0.007

Comparison of means using the one-way ANOVA test. Comparison of proportions using the chi-square test.

NA, not applicable; P_25_–P_75_, 25th percentile–75th percentile; SD, standard deviation.

General prevalence of sensitization to *P. occidentalis’* pollen in AR subjects was 21%; a light increase was observed in western and southeastern centers compared to northeastern, but without any significant difference (*P* = 0.439), (Table 2).

**Table 2.** Prevalence of pollen sensitization of *Platanus occidentalis* among adults with allergic rhinitis

	Prevalence*		
	n	%	95% CI
Total, n = 404	84	20.8	17.1–25.0
Occidental, n = 124	27	21.8	15.4–29.9
Southeastern, n = 192	43	22.4	17.1–28.8
Northeastern, n = 88	14	15.9	9.6–25.1

95% CI, 95% confidence interval.

* *P* = 0.439 between the centers. Contrast of proportions using chi-square.

Univariate analysis revealed related factors to allergic sensitivity to sycamore by a positive cutaneous test to pollens and fungi, (Table 3); unlikely, neither sex, age, duration, or symptoms severity of AR, were associated with such pollen.

**Table 3.** Univariate analysis of factors associated with pollen sensitization of *Platanus occidentalis*

	Sensitization		*P*
	Yes	No	
	n = 84	n = 320	
Age, years, media ± DE	32.2 ± 12.1	34.2 ± 13.1	0.202
Sex, n (%)			0.526
Woman	51 (60.7)	182 (56.9)	
Man	33 (39.3)	138 (43.1)	
Allergic rhinitis			
Age of onset, years, mean ± SD	14.3 ± 12.8	13.3 ± 12.2	0.512
Severity, n (%)			
Mild	11 (13.1)	52 (16.2)	0.478
Moderate-Severe	73 (86.9)	268 (83.8)	
Duration, n (%)			
Intermittent	10 (11.9)	43 (13.4)	0.711
Persistent	74 (88.1)	277 (86.6)	
Atopic comorbidity, n (%)			
Oral allergy syndrome	9 (10.7)	36 (11.2)	0.890
Allergic conjunctivitis	59 (70.2)	217 (67.8)	0.671
Allergic sensitization, n (%)			
Mites	59 (70.2)	202 (63.1)	0.225
Cockroach mix	30 (35.7)	108 (33.8)	0.735
Cat or dog epitheliums	37 (44.0)	93 (29.1)	0.009
Trees	72 (85.7)	187 (58.4)	<0.0001
Weeds	68 (81.0)	174 (54.4)	<0.0001
Grass	64 (76.2)	173 (54.1)	<0.0001
Fungi	33 (39.3)	74 (23.1)	0.003

*P* value was obtained by the *t* Student or chi-square test.

DE, standard deviation.

Multivariate analysis identified meaningful factors related to sycamore sensitization: tree pollens (OR, 3.19; *P* = 0.001), weeds (OR, 2.49; *P* = 0.004), fungi (OR, 1.96; *P* = 0.014), and cat or dog epitheliums (OR, 1.88; *P* = 0.018), (Table 4).

**Table 4.** Multivariate analysis of factors associated with allergic sensitization to *Platanus occidentalis* pollen

Allergic sensitization	Unadjusted model			Adjusted model		
	OR	95% CI	*P*	OR	95% CI	*P*
Trees	2.95	1.49–5.85	0.002	3.19	1.62–6.29	0.001
Weeds	2.26	1.20–4.26	0.011	2.49	1.34–4.64	0.004
Fungi	1.92	1.12–3.30	0.018	1.96	1.14–3.36	0.014
Cat or dog epitheliums	1.83	1.08–3.09	0.024	1.88	1.11–3.17	0.018
Grass	1.55	0.85–2.81	0.150	–	–	0.148

OR, odds ratio obtained through binary logistic regression. In the adjusted model those covariates without significant association were excluded from the model.

CI, confidence interval.

## 4. Discussion

The prevalence of allergic sensitization to *P. occidentalis* pollen is not an uncommon event in AR, as one of each 5 patients is sensible to it. This multicentric study also showed that allergic cosensitization to epitheliums, trees, weeds, and fungi increases the probability of sycamore sensitization

Twenty-five years ago, Subiza et al. [9] first described the ability of plane tree to produce allergic sensitization; they revealed that 52% of the patients challenged had a positive cutaneous test; later on, they identified a 17 kD protein as the predominant allergen in *Platanus hybrida*’s pollen, strongly related with pollinosis symptoms [17].

To date, more studies around the world have shown the ability of sycamore to produce allergic sensitization. A study in Turkey aimed to determine this frequency from pollen previously found in air, reported that 16 of 54 patients (29.1%) reacted to plane tree pollen [7]. A descriptive review of *P. occidentalis* in the Middle East showed a frequency from 1% to 57% in rhinitis or asthma cases [10]. Recently, in the same geographic area, from 602 allergic patients; 32.8% revealed a positive cutaneous test for sycamore [18].

In China, the prevalence and sensitization trend to aeroallergens for a 10-year period was tested as well, reporting a tree allergy prevalence of 7.7%, thus, assuming that allergy frequency to sycamore was relatively low [11]. Moreover, in Latin America, a study with 234 patients in Chile showed 8.1% sensitization frequency to *P. orientalis* [12]. On the other hand, evidence of this allergy prevalence to sycamore pollen was scarce in our country until now. A study evaluating allergy sensitization patterns together with weather conditions in Mexico revealed pollen from oak and ash as the most common allergens, however, sycamore pollen was not included in this analysis [19]. Recently, data analyzed in Aguascalientes, Mexico including 350 patients with AR aimed to establish sensitization frequency to local allergens; found that pollens from trees were the ones with more sensitivity for patients (65%), and although sycamore was tested, the number of subjects with a positive result to such pollen was not mentioned [20]. Our results widely differ from 2 trials carried out in Mexico, one of them in the pediatric population in Mexico City and the second at Hospital Universitario de Monterrey; which reported sensitization prevalence of *P. orientalis* and *P. occidentalis* in 1.8% and 6.7%, respectively [21, 22]. Our results indicating sensitization frequency to *P. occidentalis*’ pollen, agree with international data, then, considering their national representativeness as they came from 3 different geographical centers in Mexico, we could highly advise the inclusion of this pollen to assess patients with allergic diseases.

Associated factors related to the probable increase of allergic sensitivity to *P. occidentalis* included a cosensitization from tree pollen. Even though the main allergen of *P. occidentalis* has been properly purified (*Pla oc* 1), this molecule might not be responsible of such a relationship; it belongs to the group of invertase inhibitor proteins and shares structural homologies with other invertases from other plane tree species (*Pla a* 1, *Pla or* 1 and *Pla r* 1) [23]. Instead, profilins (*Pla a* 8) or lipid transporter proteins (*Pla a* 3) identified in other plan tree species, would explain such association, even more if these proteins are expressed in all plane tree species. Besides, a molecule forms the group of thaumatins (*Pla a* 8) might also be a candidate to explain the sensitivity to weeds and its relation with sycamore [23]. Other molecules as *Amb a* 1 and *Art v* 6, from ambrosia and mugwort respectively, and *Pru p* 3 have shown homologies with *Pla a* 3 as well [24].

About sycamore´s relation with epitheliums and fungi, no biological explanation of this relationship exists until now.

Limitations of the study included identification of allergic sensitization as aeroallergens were not standardized for each participating center; consequently, it was not possible to identify which pollen from trees or weeds was closely related to *P. occidentalis* sensitization. On the other hand, even when sensitization frequency of sycamore was considerable, this fact does not necessarily indicate that patients’ symptoms were triggered by exposition to this pollen. Another limitation is related to species’ diversity within the gender *Platanus;* more studies are required to assess if allergic sensitization prevalence to sycamore local species is similar to the one observed in the present study, where a non-native Mexican species was used. Moreover, as plane tree is commonly used to decorate big cities rather than rural zones, its sensitization prevalence is unknown among localities.

It is also necessary to consider the time in which patient enrollment was carried out, which took place during the months of August to March; in Mexico, a local species of sycamore, *P. mexicana*, tends to flower during the months of December to April [25]. Finally, no pediatric population was included in our study, thus, conclusions in this age group were not possible.

In conclusion, allergic sensitization to trees, weeds, fungi, and epitheliums of cats or dogs is associated with *P. occidentalis* sensitization, which is common in Mexico in patients with AR. It is advisable for allergologists who receive this kind of patient to include sycamore pollen in nasal allergy evaluations.

## Conflicts of interest

The authors have no financial conflicts of interest.

## Authors contributions

Conception and design: Martín Bedolla-Barajas, Luis Alfredo Jiménez-Huerta, and Angie Bedolla-Pulido. Provision of study materials or patients: Martín Bedolla-Barajas, Javier Domínguez-Morales, and Ilse Mariana Loya-Barriga. Analysis and interpretation of the data: Jaime Morales-Romero and Martín Bedolla-Barajas. Statistical expertise: Jaime Morales-Romero. Critical revision of the article for important intellectual content: Martín Bedolla-Barajas, Javier Domínguez-Morales, and Ilse Mariana Loya-Barriga. Final approval of the article: Martín Bedolla-Barajas, Javier Domínguez-Morales, Ilse Mariana Loya-Barriga, Angie Bedolla-Pulido, Luis Alfredo Jiménez-Huerta, and Jaime Morales-Romero.
